# Guillain-Barré syndrome triggered by surgery in a Chinese population: a multicenter retrospective study

**DOI:** 10.1186/s12883-021-02067-1

**Published:** 2021-01-28

**Authors:** Qiaoyu Gong, Shuping Liu, Yin Liu, Jiajia Yao, Xiujuan Fu, Zheman Xiao, Zuneng Lu

**Affiliations:** Department of Neurology, Renmin hospital of Wuhan University, 99 Zhang Zhidong Road, Wuchang District, Wuhan, 430060 Hubei Province PR China

**Keywords:** Clinical features, Disease severity, Guillain-Barré syndrome, Prognosis, surgery.

## Abstract

**Background:**

Surgery is a potential trigger of Guillain-Barré syndrome (GBS), a disorder which leads to an autoimmune-mediated attack of peripheral nerves. The present study was designed to explore clinical features of post-surgical GBS compared with those of general GBS in order to provide better clinical advice to patients undergoing surgery.

**Methods:**

The medical records of GBS patients who were seen at 31 tertiary hospitals in southern China between January 1, 2013 and September 30, 2016 were retrospectively analyzed. Post-surgical GBS was defined as symptoms of GBS within 6 weeks after surgery. Clinical features of post-surgical GBS are described and are compared with general GBS.

**Results:**

Among the 1001 GBS patient cases examined in this study, 45 (4.5%) patient cases exhibited symptoms of GBS within 6 weeks of undergoing surgery. Within this group, 36 (80.0%) patients developed initial symptoms of limb weakness. The average interval between surgery and symptom onset was 13.31 days. The most common type of surgery which triggered GBS was orthopedic surgery, followed by neurological surgery. Compared to general GBS, post-surgical GBS was characterized by a higher proportion of severe patients (Hughes functional grading scale (HFGS) score ≥ 3) upon admission and at nadir, higher HFGS scores at discharge, and longer hospital stays. Post-surgical GBS patients also had a significantly higher frequency of the acute motor axonal neuropathy subtype (37.9 vs. 14.2, respectively; *P* = 0.001).

**Conclusion:**

Surgery is probably a potential trigger factor for GBS, especially orthopedic surgery. Infections secondary to surgery may play a role. The possibility of preceding (post-operative) infections was not excluded in this study. Clinical presentation of post-surgical GBS is characterized by a more severe course and poorer prognosis, and should be closely monitored.

**Trial registration:**

chicTR-RRc-17,014,152.

## Background

Guillain-Barré syndrome (GBS) is an immune-mediated acute polyradiculoneuropathy which is characterized by rapidly progressive muscular weakness and hyporeflexia or areflexia [1]. Antecedent infections 6 weeks prior to symptom onset are present in approximately 2/3 of GBS patients [2]. In addition, non-infectious factors such as vaccinations, trauma, and surgery have been reported as possible triggers of GBS. Several case reports have also described the occurrence of GBS following cardiac surgery, gastrointestinal surgery, neurological surgery, orthopedic surgery, and laparoscopic prostatectomy [3–15]. Gensicke et al. reported that the incidence of GBS after surgery was significantly higher than for GBS triggered by infection or vaccine [16]. Thus, surgery has been identified as a potential risk factor for GBS [17]. In a study conducted by Li et al., all nine patients exhibited an axonal, rather than demyelinating, form of neuropathy [18]. Moreover, Hocker et al. reported that post-surgical GBS was more common in patients with an active malignancy [19]. Among 17 post-surgical GBS patients and 66 non-surgical GBS patients in another study, the former group exhibited severe motor dysfunction and poor prognosis [20]. However, previous studies have been limited by small sample sizes. As a result, the clinical features of post-surgical GBS are not well understood. Thus, it remains unclear whether post-surgical GBS results in a more severe course or poorer prognosis compared with GBS triggered by other risk factors. It is also unclear whether patients should be more concerned about particular types of surgery.

To date, these considerations have not been explored systematically in a large cohort of GBS patients. Therefore, we conducted a multicenter retrospective study to explore the clinical features of post-surgical GBS and to identify those that provide an early clinical warning or could indicate the need for early intervention.

## Methods

### Patients

The medical records of consecutive patients hospitalized with a diagnosis of GBS in 31 representative tertiary hospitals, located in 14 provinces in southern China, between January 1, 2013 and September 30, 2016 were retrospectively analyzed [21]. Three neurologists and 12 well-trained GBS team members at the Department of Neurology, Renmin Hospital of Wuhan University (Wuhan, China), were involved in the confirmation of diagnosis for each GBS patient [21]. Details regarding those members are described in our previous study [21]. The patients who met the established clinical criteria of Asbury and Cornblath (1990) [22] were included in this study. For the patients whose diagnosis was in doubt, a comprehensive consideration of both clinical presentation and ancillary data was made before including their medical records for analysis. Patients who received an alternative diagnosis of weakness and those who abandoned examination and treatment within 5 days after admission were excluded [21]. Details regarding clinical data extraction and analysis are described in our previous study [21].

This study was approved by the Ethics Committee of Renmin Hospital of Wuhan University. The need for informed consent was waived.

### Variables

Clinical data that were collected included patient age, gender, antecedent infections, history of diabetes mellitus and hypertension, initial symptoms, Hughes functional grading scale (HFGS) score upon admission, time between surgery and symptom onset, type of surgery, cerebrospinal fluid, HFGS score at nadir, duration of hospitalization, HFGS score at discharge, requirement for mechanical ventilation, modality, electrodiagnostic subtypes, and treatment. Electrodiagnostic criteria proposed by Hughes [23] (see online Supplementary Table) were used to define GBS subtypes in patients with available electrophysiological data. Post-surgical GBS was defined as symptoms of GBS within 6 weeks after surgery.

Clinical features of post-surgical GBS were recorded and compared with clinical features of GBS without recent surgery.

### Evaluation of disease severity and short-term prognosis

HFGS score [24], a widely accepted scale of disability for GBS, was applied. This scale includes: grade 0, healthy; grade 1, minor signs or symptoms of neuropathy but capable of manual work; grade 2, able to walk without support of a stick but incapable of manual work; grade 3, able to walk with a stick, appliance, or support; grade 4, confined to bed or chair bound; grade 5, requiring assisted ventilation; grade 6, death due to GBS. A severe form of disease was defined as an HFGS score above or equal to 3 upon admission or at nadir, while a good short-term prognosis was defined as an HFGS score less than 3 at discharge.

### Statistical analysis

Statistical analysis was performed by using SPSS V.25.0 software (IBM, West Grove, PA, USA). Categorical data were reported as proportions. Continuous data exhibiting a normal distribution were presented as mean ± standard deviation (SD) and were analyzed with Student’s *t*-test. Continuous data not exhibiting normal distribution were reported as median and interquartile range (IQR) values and were analyzed with the Mann-Whitney U-test or the Wilcoxon signed rank test. Chi-square and Fisher exact tests were used to determine whether differences in proportions were significant. *P*-values less than 0.05 were considered significant.

## Results

The medical records of 1001 GBS patients were examined in this retrospective study. These cases involved 601 males and 400 females with a mean age of 51.48 y. Forty-five (4.5%) patients exhibited symptoms of GBS within 6 weeks of undergoing surgery and were identified as having post-surgical GBS.

### Characteristics of post-surgical GBS patients

**Fig. 1:**
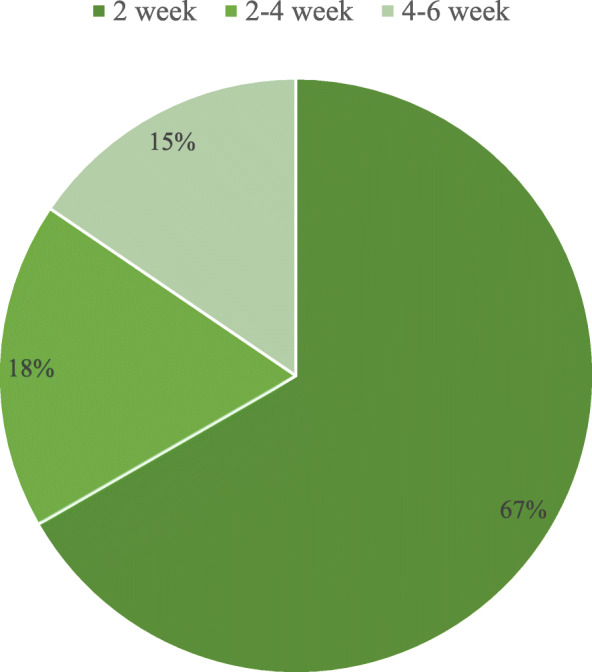
A bar graph of the interval between surgery and GBS symptoms

Demographic characteristics and clinical features of the post-surgical GBS patients (*n* = 45) are presented in Table 1. This group consisted of 19 females (42.2%) and 26 males (57.8%) with a mean age of 53.93 ± 12.86 y (range: 24–79). The average interval between surgery and symptom onset was 13.31 d (range: 1–40). For 67% of these patients, symptoms of GBS appeared within 2 weeks after surgery (Fig. 1). In 36 (80.0%) patients, initial symptoms of motor weakness were observed, while 12 (25.2%) patients developed initial symptoms of sensory change. There were 11 (24.4%) patients with a history of hypertension and 4 (8.9%) patients with a history of diabetes mellitus. Lumbar puncture was performed for 31 patients, among which 23 patients presented albumino-cytological dissociation. According to electrophysiological criteria, patients were diagnosed with acute inflammatory demyelinating polyneuropathy (AIDP) (*n* = 21), acute motor axonal neuropathy (AMAN) (*n* = 11), acute motor sensory axonal neuropathy (AMSAN) (n = 2), equivocal results (*n* = 3), and 1 was normal. HFGS score was determined to assess clinical severity and prognosis. The proportion of severe patients (HFGS score ≥ 3) upon admission, or at nadir, was 82.2 and 91.1%, respectively. The average HFGS score at discharge was 2.78 (Table 1). All of the patients were treated actively after admission. Eighteen patients were treated with intravenous immunoglobulin, 10 with glucocorticoid, 15 with a combination of intravenous immunoglobulin and glucocorticoid, 1 with plasmapheresis, and 1 with a combination of glucocorticoid and plasmapheresis.

**Table 1:** Demographic characteristics and clinical features of the post-surgical GBS and general GBS patients examined in this study

Parameters	GBS without recent surgery (*n* = 956)	GBS after surgery (*n* = 45)	Two-tailed *P*-value
Patient age (mean, y)	51.37 ± 15.85	53.93 ± 12.86	0.285
Male, n (%)	575 (60.1)	26 (57.8)	0.751
Diabetes mellitus, n (%)	72 (7.5)	4 (8.9)	0.962
Hypertension, n (%)	241 (25.2)	11 (24.4)	0.908
Initial symptoms, n (%)			
Motor weakness	729 (76.3)	36 (80.0)	0.563
Sensory change	402 (42.1)	12 (25.2)	**0.041**
HFGS score upon admission, n (%)			
≥ 3	608 (63.6)	37 (82.2)	**0.011**
< 3	348 (36.4)	8 (17.8)	
HFGS score at nadir, n (%)			
≥ 3	692 (72.4)	41 (91.1)	**0.006**
< 3	264 (27.6)	4 (8.9)	
Hospital stay (d)	15 (11–20)	21 (14–26)	**0.000**
HFGS score at discharge (g)	2 (1–3)	3 (2–4)	**0.001**
Mechanical ventilation (MV), n (%)	91 (9.5)	4 (8.9)	1.000
Deaths during hospital stay, n (%)	6 (0.6)	1 (2.2)	0.276
Lumbar puncture	757	31	
Mean protein concentration (g/L)	1.18 ± 0.86	0.99 ± 0.71	0.346
Albumin-cytologic dissociations, n (%)	611 (80.7)	23 (74.2)	0.370
Electrodiagnostic subtypes, n (%)	691	29	
AIDP	339 (49.1)	12 (41.4)	0.418
AMAN	98 (14.2)	11 (37.9)	**0.001**
AMSAN	21	2	0.236
Treatment, n (%)			
IVIg	407 (42.6)	18 (40.0)	0.733
Plasmapheresis	41 (4.3)	1 (2.2)	0.768

*AIDP* acute inflammatory demyelinating polyneuropathy, *AMAN* acute motor axonal neuropathy, *AMSAN* acute motor sensory axonal neuropathy, *GBS* Guillain-Barré syndrome, *HFGS* Hughes Functional Grading Scale, *IVIg* intravenous immunoglobulin. Statistically significant results are shown in bold

### Categories of surgeries

Details of the surgeries performed are presented in Table 2. The most common type of surgery triggering GBS was orthopedic surgery, with a proportion of 46.7%. This was followed by neurological surgery (17.8%), ophthalmic surgery and gastrointestinal surgery (8.9%), cesarean delivery (6.7%), coronary bypass surgery (4.4%), and artificial abortion, thoracic surgery, and vocal cord polyp resection (2.2%).

**Table 2:** Categories of the surgeries performed (n = 45)

Type of surgery	n (%)
**Orthopedic surgery**	**21 (46.7)**
Fracture	9
Lumbar intervertebral disc	6
Lumbar stenosis	1
Lumbar spondylolisthesis	1
Cervical internal disc herniation	1
Hemangioma of the phalanx	1
Foot injury	1
Bone graft	1
**Neurological surgery**	**8 (17.8)**
Subdural hematoma	2
Hydrocephalus	2
Meningioma	1
Hypophysoma	1
Cerebral aneurysm	1
Trifacial neuralgia	1
**Ophthalmic surgery**	**4 (8.9)**
Glaucoma	1
Retinal detachment	1
Conjunctival melanoma	1
Ocular trauma	1
**Gastrointestinal surgery**	**4 (8.9)**
Gastric polyps	1
Esophageal carcinoma	1
Appendicitis	1
Cholecystitis	1
**Cesarean delivery**	**3 (6.7)**
**Artificial abortion**	**1(2.2)**
**Coronary bypass surgery**	**2(4.4)**
**Thoracic surgery**	**1(2.2)**
**Vocal cord polyp resection**	**1(2.2)**

### Comparison of disease severity and prognosis between GBS patients with and without surgery

Compared with GBS patients without recent surgery, patients developing GBS after surgery were characterized by a higher proportion of severe patients (HFGS ≥3) upon admission (82.2 vs. 63.6, respectively; *P* = 0.011) and at nadir (91.1 vs. 72.4, respectively; *P* = 0.006) (Table 1). In addition, a higher HFGS score at discharge (3 vs. 2, respectively; *P* = 0.001) and longer hospital stays (21 d vs. 15 d, respectively; *P* = 0.000) were observed. The post-surgical GBS patients also had a significantly higher frequency of the AMAN subtype (37.9 vs. 14.2, respectively; P = 0.001), yet a significantly lower frequency of developing initial symptoms of sensory change (25.2 vs. 42.1, respectively; *P* = 0.041). There were no significant differences between the other variables.

## Discussion

In this multicenter retrospective study of 1001 GBS patients, it was observed that surgery, especially orthopedic surgery, was probably an important preceding event of GBS. Furthermore, post-surgical GBS presented a more severe course and poorer prognosis compared with GBS triggered by other risk factors. Thus, surgery probably represents a risk factor for GBS which should not be overlooked.

Previous studies have reported the occurrence of post-surgical GBS to be 9.1% [19], 6.6% [25], 19.4% [26], 5.8% [27], and 9.5% [16]. In the present study, the rate of post-surgical GBS among 1001 patients was 4.5%. This lower rate may be due to the small sample size of previous studies. It has also been observed that post-surgical GBS exhibits a male preponderance, with a gender ratio of 3.5 between male and female patients **Error! Reference source not found.**. However, no gender differences were observed in the present study compared with general GBS patients. Regarding the effect of age on morbidity, disagreement arose when post-surgical GBS was compared with general GBS. A literature review revealed that post-surgical GBS occurs mostly between the ages of 50 and 70 years [17]. In contrast, Sejvar et al. reported two peaks in cases of general GBS, with the first among young adults (20–30 y) and a second peak among the elderly (> 60 y) [28]. In the present study, the mean age of our post-surgical GBS patients was 53.93 y, and there was no age difference with the general GBS patients. Thus, it remains unclear whether older individuals are more likely to develop GBS after surgery. It is possible that the age distribution for post-surgery GBS compared to that for general GBS may not be meaningful unless it is adjusted for the distribution of ages for all surgical patients at the same time. It is also possible that the population more likely to undergo surgery (> 50 y) may influence the findings.

Surgical operations can cause immunosuppression in the post-operative period. The degree or duration of immunosuppression is often determined by the magnitude of the initial surgical insult [29]. An immunosuppressive effect has been found to be strongest on the third day after surgery, and it subsequently recovered between days 7 and 10 [17]. These observations are consistent with the manifestation of GBS symptoms within 2 weeks after surgery in 67% of our cohort. In addition, 36 out of 45 (80.0%) post-surgical GBS patients in the present study initially presented with limb weakness. Therefore, patients who exhibit unexplainable symptoms of symmetrical limb weakness within 2 weeks after surgery should be closely monitored for GBS.

Orthopedic surgery was the most common type of surgery which triggered GBS, followed by neurological surgery. The latter surgeries are prone to causing neurologic complications, including spinal cord ischemia, spinal cord hemorrhage, cauda equina syndrome, direct injury to nerves, and epidural abscesses [17]. Clinical manifestation and required physical examination or imaging data can be used to determine a diagnosis. We validated a post-surgical GBS diagnosis for each of the cases examined here. Furthermore, each of these cases presented typical clinical manifestations and signs of GBS.

To date, the underlying pathogenesis of post-surgical GBS is not fully understood. There are several mechanisms which have been proposed. For example, a sensitizing mechanism involving release of antigens and subsequent antigen autoimmunity due to surgery may contribute to the development of GBS [30]. Immunosuppression induced by surgery may result in autoantibody-mediated attacks of peripheral nerves during the post-operative period. Correspondingly, post-surgical patients have an elevated risk of infection due to immunosuppression. For example, a previous study reported that 42.8% of post-surgical GBS patients developed infections [26]. Endocrine stress systems may also be activated by surgery, resulting in hypersecretion of adrenocorticotropic hormone and imbalance of the immune system [17, 31–33]. Surgical trauma/traction, tourniquet pressure, and malposition of the patient which apply pressure to nerves can also increase susceptibility to nerve damage. Local trauma to nerves can potentially create conditions for interactions between the immune system and myelin, which can further trigger a cascade of immunologic events. During surgery, various instruments and devices, such as retractors, can compress nerves directly [34]. Instruments and devices are used more often in orthopedic surgery, and this could explain why this type of surgery is more prone to triggering GBS. Furthermore, administration of epidural anesthesia before surgery, particularly for orthopedic and neurological surgeries can also potentially induce breakdown of the blood-brain barrier. Compromise of this barrier could allow central immunogenic factors to enter the peripheral nervous system; although these factors may only mediate a weak immunopathogenic effect on the peripheral nervous system. The latter hypothesis is consistent with the lower incidence of GBS triggered by orthopedic or neurological surgeries.

AIDP and AMAN are two important subtypes of GBS. In a previous study, 15/17 post-surgical GBS patients were diagnosed with AMAN, while the remaining two with AMSAN [20]. In the present study, the post-surgical GBS patients exhibited a significantly higher frequency of the AMAN subtype and a significantly lower frequency of developing initial symptoms of sensory change compared with the GBS patients without recent surgery. These results indicate that post-surgical GBS patients are more likely to experience axonal damage. However, as a retrospective study, sufficient electrophysiological data were not available for all of the post-surgical GBS patients. Thus, further studies are needed to more completely characterize the electrophysiological features of post-surgical patients. It has also been observed that patients with the AMAN subtype frequently have serum antibodies against GM1a, GM1b, GD1a, and GalNAc-GD1a gangliosides [1, 2]. Such antibodies that target ganglioside complexes can promote complement activation and induce peripheral nerve injury [2]. In the present study, information regarding serum antibodies against GM1a, GM1b, GD1a, and GalNAc-GD1a gangliosides was lacking in most of the post-surgical GBS cases. Thus, additional studies also need to confirm the distribution of anti-ganglioside antibodies in post-surgical patients.

Post-surgical GBS patients can manifest severe motor dysfunction, they have a high risk of respiratory failure, and they often receive a poor prognosis [20]. Correspondingly, our results revealed higher HFGS scores, independent of their collection upon admission, at nadir, or at discharge, in post-surgical GBS patients. These scores indicate remarkable increases in disease severity and adverse short-term outcomes, and may be explained as follows. Patients with an axonal form of GBS presented more severe clinical courses and poorer prognosis compared to patients with the demyelinating form of GBS. For these patients, stress and traumas induced by surgery were additional setbacks to recovery from disease. Furthermore, we found no significant between-group differences in terms of mechanical ventilation and death during their hospital stay. Possible bias exists that patient functioning after surgery may influence GBS disability scores. For some patients undergoing surgery, physical ability is limited. It is not easy to affirm whether the severity of limb weakness was purely related to GBS.

There were limitations associated with the present study. First, as a retrospective study, the total number of surgical cases during the time period examined was not available. These data could have served as a denominator for the incidence of post-surgical GBS. Second, information was also not available regarding patient functioning after surgery. These data are needed in future studies and could be helpful in evaluating GBS disability scores. Third, information regarding long-term follow-up of post-surgical GBS patients was lacking. Fourth, information regarding complications of surgery, especially in regard to infection, is important. These data could provide further insight into the underlying pathogenesis of post-surgical GBS. Complications may also influence the functional status of a patient and disease severity. In the present study, details regarding the surgeries performed were lacking. However, despite these limitations, a notable strength of the present study was the large sample size of this multi-center study. This aspect guaranteed the reliability of our observations.

## Conclusions

The results of the present study indicate that surgery is probably a potential trigger factor for GBS, especially orthopedic surgery. In addition, the clinical presentation of post-surgical GBS was characterized by a more severe course and a poorer short-term prognosis. These patients mainly exhibited weakness symptoms within 2 weeks after surgery. Consequently, when postoperative patients report unexplained progressive muscle weakness, GBS should be noted. If confirmed, appropriate treatment should be provided to alleviate symptoms as soon as possible. If necessary, physicians should transfer patients with GBS to an intensive care unit. An early diagnosis can help initiate appropriate treatment and potentially improve patient prognosis.

## Data Availability

The datasets generated and/or analyzed during the current study are not publicly available due to institutional restrictions (http://www.gov.cn/gongbao/content/2017/content_5227817.htm). The corresponding author has permissions to access the raw data and the datasets are available from the corresponding author on reasonable request.

## Abbreviations

GBS: Guillain-Barré syndrome
HFGS: Hughes Functional Grading Scale
AIDP: Acute inflammatory demyelinating polyneuropathy
AMAN: Acute motor axonal neuropathy
AMSAN: Acute motor sensory axonal neuropathy
SD: Standard deviation
